# Pharmacological basis of the antifibrotic effects of pirfenidone: Mechanistic insights from cardiac *in-vitro* and *in-vivo* models

**DOI:** 10.3389/fcvm.2022.751499

**Published:** 2022-09-20

**Authors:** Laura Sartiani, Gianluca Bartolucci, Marco Pallecchi, Valentina Spinelli, Elisabetta Cerbai

**Affiliations:** ^1^Section of Pharmacology, Department of Neurosciences, Psychology, Drug Research and Child Health (NEUROFARBA), University of Florence, Florence, Italy; ^2^Section of Medicinal Chemistry, Department of Neurosciences, Psychology, Drug Research and Child Health (NEUROFARBA), University of Florence, Florence, Italy

**Keywords:** pirfenidone, cardiac fibrosis, myofibroblasts, TGF-β, hypertrophy

## Abstract

Pirfenidone is a small drug with marked antifibrotic activity approved for the treatment of Idiopathic pulmonary fibrosis. Recently, its peculiar pharmacological profile has attracted attention for its potential therapeutic benefit for extra-pulmonary disorders characterized by pathological fibrosis, such as kidney, liver, and cardiac failure. A major pitfall of pirfenidone is the lack of consistent understanding of its mechanism of action, regardless of the target. In addition to the increasing attention to the role of inflammation and its mediators in several processes, a better knowledge of the variety of fibroblasts' population, of signals controlling their activation and trans-differentiation, and of crosstalk with other cell resident and non-resident cell types is needed for prevention, treatment and possibly reverse of fibrosis. This review will focus on pirfenidone's pharmacological profile and its effects on cardiac fibroblasts.

## Introduction

Pirfenidone has been approved for the treatment of Idiopathic Pulmonary Fibrosis in Japan, the European Union (EMA), Canada, and the United States (FDA). Recently, due to its peculiar pharmacological profile and mechanism of action, this drug has attracted attention and has been proposed for extra-pulmonary disorders characterized by pathological fibrosis, such as chronic kidney, liver and cardiac diseases. This review will focus on pirfenidone's pharmacological profile and its effects on cardiac fibroblasts while addressing previous systematic reviews for other areas of interest (1–3).

## Pharmaceutical and pharmacokinetic properties of pirfenidone

### Drug characteristics

Pirfenidone (5-methyl-1-phenyl-2-1(H)-pyridone) is a small molecule with a molecular weight of 185.23 g/mol. Commercially, it is available as an orally administrative drug under the trade names Pirespa (Shionogi), Esbriet (Roche), and Etuary (GNI group) in the form of film-coated tablets or capsules with different dosages.

The initial dose titration (1–7 days) is 267 mg t.i.d (801 mg/day); the following week (8–14 days), this is increased to 534 mg t.i.d. (1,602 mg/day) until a maintenance dosage of 801 mg t.i.d (2.4 g/day) is reached after 15 days of treatment. The administration of pirfenidone is suggested to occur in a fed state to avoid adverse reactions such as nausea, vomiting, gastroesophageal reflux, etc. If patients experience significant adverse events (i.e., gastrointestinal, photosensitivity reaction, rash), temporary dosage reductions or therapy interruptions of pirfenidone should be considered to allow for resolution of symptoms, otherwise discontinue, if symptoms persist despite these interventions (1, 4). Pirfenidone is also used for topical treatment of patients with skin ulcers, wounds, or burns, showing continuous statistically significant scar regression, without serious adverse events (5, 6).

### Pharmacokinetic properties

The oral administration of pirfenidone is suggested in a fed state to avoid the side effects such as nausea and vomiting, although the presence of food can slightly diminish drug bioavailability. In fact, a study reports that after administration of a single oral dose of pirfenidone 801 mg for a healthy volunteer (aged 50–66 years), the area under the plasma concentration-time curve (AUC) for pirfenidone in a fed state was approximately 80–85% of the AUC for pirfenidone in a fasted state. The lack of bioavailability after eating is related to the reduction of C_max_ (on average 50%) reached in 3.5 h, with respect to a T_max_ value of 0.5 h in a fasted state. In human beings, the adsorbed pirfenidone binds for 50–58% to serum albumin, while the remaining is solubilized in the plasma. In therapeutic steady state, the apparent volume of distribution is about 70 L (1–4).

### Metabolism and elimination

Pirfenidone is largely metabolized by the liver, mainly *via* CYP1A2, into the pharmacologically active metabolite 5-carboxy-pirfenidone, which is in turn eliminated by glomerular filtration (80%) within 24 h of oral administration. The half-life and clearance of pirfenidone, after the administration of a single dose of 801 mg, are between the 2.4–2.9 h and 13.8–11.8 L/h respectively. In general, no dose adjustment is required in the elderly, but careful titration is required in those with mild hepatic insufficiency and is contraindicated in patients with severe hepatic failure or liver diseases. Attention should also be paid to drug–drug interactions, especially with CYP1A2 inhibitors or substrates, such as propafenone and amiodarone. Cigarette smokers may have reduced bioavailability of pirfenidone, hence less efficacy, due to CYP1A2 induction and increased drug metabolism and clearance.

## Pharmacological properties

The mechanism of action of pirfenidone has been clarified only in part; nevertheless, a deeper understanding of its molecular features is an essential premise to a wider indication for diseases characterized by extensive fibrosis such as heart failure and hypertrophic cardiomyopathy (7, 8). For this reason, it is essential to have a brief excursus on the mechanisms underpinning cardiac fibrosis.

### Cellular and molecular mechanisms of cardiac fibrosis

**Figure 1:**
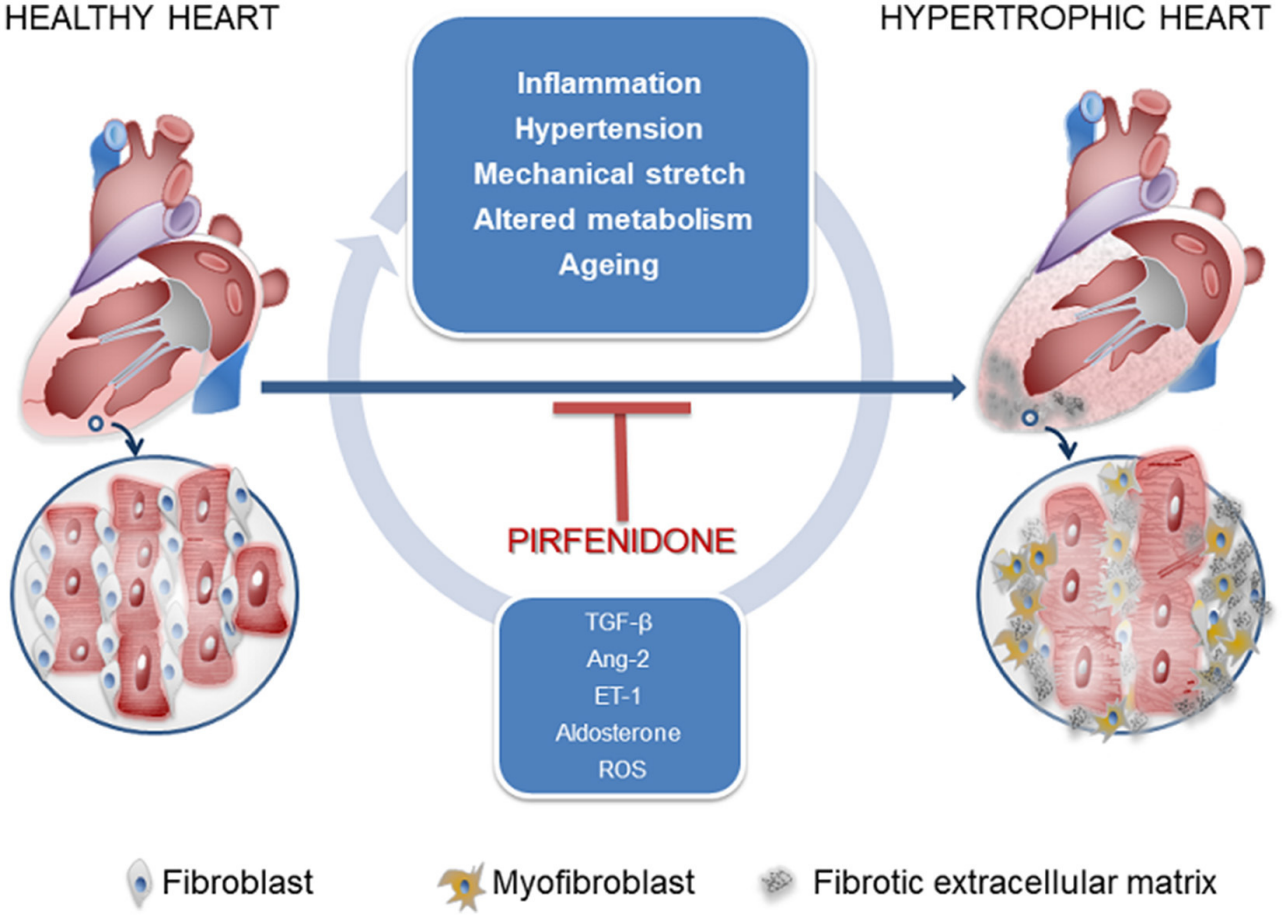
Signals controlling fibroblast-to-myofibroblast transdifferentiation and favoring cardiac interstitial fibrosis.

Cardiac fibrosis consists of the deposition of a collagen matrix by fibroblasts (Figure 1). Reparative fibrosis is essential for healing the injured myocardial tissue after infarction and preventing fibroblast activation causes inefficient collagen production and ventricular rupture (9). However, interstitial fibrosis may diffuse amid cardiomyocytes and around blood vessels and may generate a thick extracellular matrix, which impairs normal cardiac function. Such fibrosis is one of the consequences of maladaptation of the heart to a variety of noxious stimuli, such as inflammation, hypertension, mechanical stretch, circulating cytokines, altered metabolism, and aging. Trans-differentiation of fibroblasts into myofibroblasts (MyoFb) plays a major role in cardiac fibrosis, since MyoFb are secretory cells, producing collagen and matrix components that can form scar tissue but also cytokines in a vicious circle that amplifies fibrosis. The most powerful trigger of this process is transforming growth factor-beta (TGF-β) (10). TGF-β1 acts as a paracrine factor released by cells of the immune systems, in particular, macrophages, which appear early when a protective or reparative response is required: TGF-β1, in fact, also promotes numerous biological pathways and holds anti-inflammatory properties. For its properties, TGF-β1 is one of the targets of antifibrotic agents as monoclonal antibodies (GC1008, LY2382770).

From an embryological point of view, cardiac fibroblasts have no unique progenitors but mature cells possess specific markers: discoidin domain-containing receptor 2 (DDR2), platelet-derived growth factor receptor-α (PDGFR-α), and transcription factor 21 (Tcf21) (11). During cardiogenesis, the epicardium is a major source of non-myocardial cell types in the heart and contributes to most of the fibroblast population; other sources of fibroblasts are the endocardium (e.g., in the interventricular septum) and the neural crest (e.g., in the right atrium). Fibroblasts in the developing heart produce periostin, not in the adult heart if quiescent; conversely, MyoFib involved in cardiac remodeling express α-smooth muscle actin (α-SMA) and periostin (12), a sort of de-differentiation toward a fetal phenotype allowing for active proliferation and matrix deposition.

**Figure 2:**
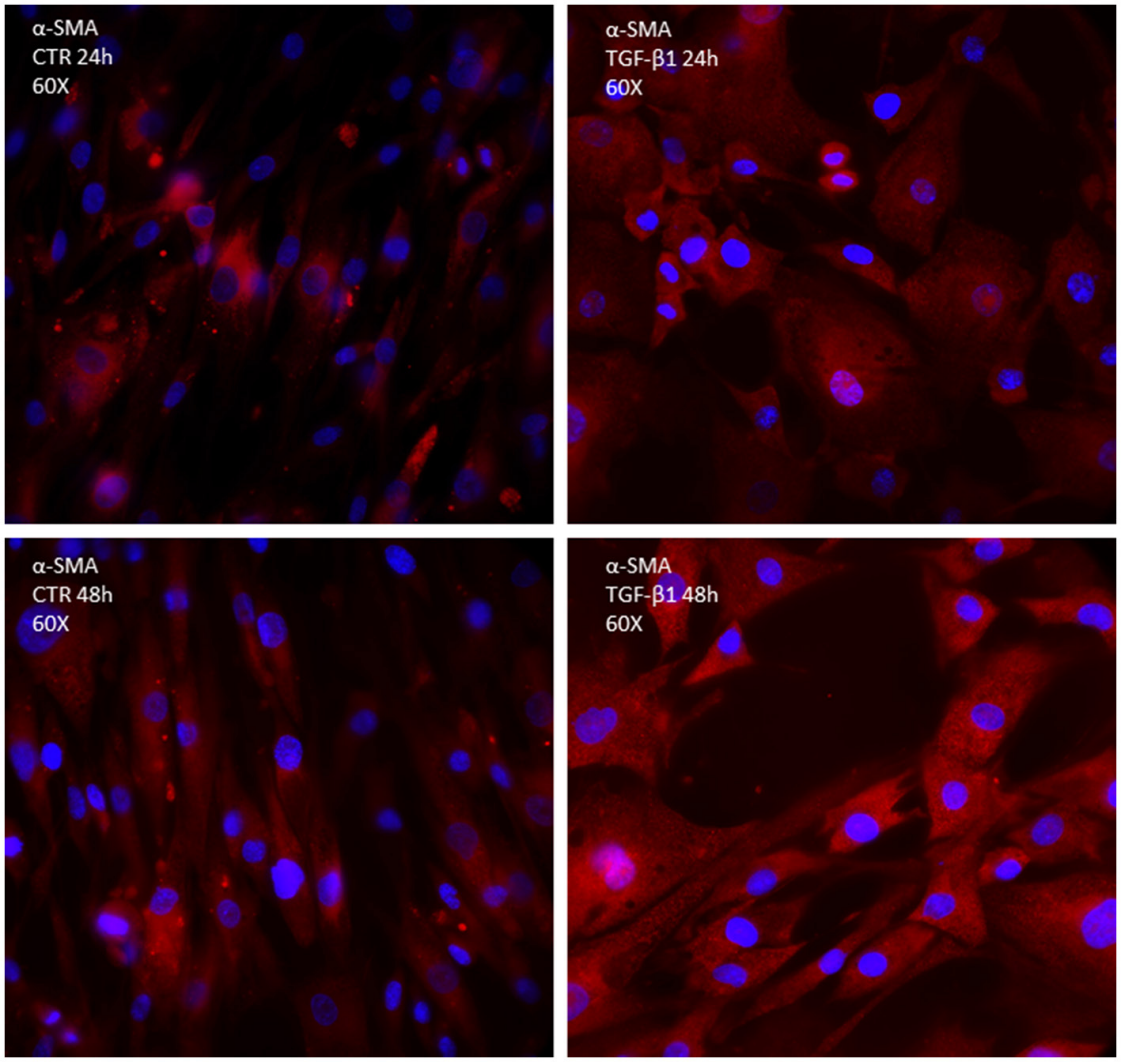
Immunocytochemistry of primary culture of fibroblasts isolated from human biopsies of patients undergoing cardiosurgery for obstructive human hypertrophic cardiomyopathy; α-SMA is marked in red; DAPI is marked in blue.

Transforming growth factor-beta binds to the type I and II receptors, which activate the so-called canonical Smad3/4 pathway, transcription factors essential for promoting the synthesis of procollagen III and interstitial fibrosis; however, it seems important for healing and premature inhibition of reparative scar predisposes to dilation and rupture in infarcted murine hearts (13). TGF-β also activates the non-canonical p-TAK and type-4 NADPH oxidase (NOX4) pathways (Figure 2). NOX4 activation (translocation) produces reactive oxygen species (ROS), which possibly cooperate with p-TAK to promote phosphorylation of downstream signaling such as c-JNK and p-38. The final step consists of the increased trans-differentiation into the proliferative phenotype, MyoFibs, expressing alpha-smooth muscle actin (α-SMA) and secreting collagen and connective tissue growth factor (CCN2) (14). Figure 2 shows the expression of α-SMA in fibroblasts isolated from myectomies of patients undergoing surgery for obstructive hypertrophic cardiomyopathy (HCM), in the absence and presence of exposure to TGF-β for 24 and 48 h. The possibility to test the drug in patients' derived cells might allow a comparison of its properties in normal vs. diseased conditions.

### Molecular targets of pirfenidone: *In-vitro* models

*In-vitro* studies of fibroblasts and trans-differentiation have partially elucidated the properties of pirfenidone.

Little experimental evidence based on the conventional 2D culture of rodent primary cardiac fibroblasts (15) has demonstrated that pirfenidone dose-dependently decreases markers of spontaneous fibroblast-to-myofibroblast trans-differentiation, including cell proliferation, α-SMA expression, and collagen contractility. Additionally, in the same context pirfenidone reduces fibroblast migration ability as well as synthesis and secretion of TGF-β1, thus substantially replicating the effects observed or hypothesized in *in-vivo* studies. Despite being informative, such an experimental approach involving primary cardiac fibroblasts in culture does not facilitate a deeper understanding of the mechanisms and factors involved in cardiac fibrogenesis or in the protection given by potential antifibrotic agents. In fact, cultured primary fibroblast obtained from humans or animal models display elevate phenotype plasticity and sensitivity to mechanical stimuli, which intrinsically promote their trans-differentiation into myofibroblast within a few hours of plating (16, 17). At least part of this spontaneous phenotype switch is caused by marked non-physiological conditions that are present in conventional 2D culture, including the elevated stiffness of plating surfaces compared to native cardiac tissues and the occurrence of a variety of pro-fibrotic stimuli arising from culture passaging. Altogether, these conditions create a multitude of basal fibrogenic responses in the model system, which hamper physiologically pertinent studies, data reproducibility, and translational values of results.

Recently, substantial advancements in 2D *in-vitro* models of cardiac fibrosis have been obtained using human induced pluripotent stem cells (hiPSCs), which overcome the limited availability of human primary cardiac cells and the challenges to propagate them long-term *in-vitro*. Taking advantage of the ability of this cell source to appropriately model healthy and diseased human heart tissues *in-vitro*, Zhang and coworkers (18, 19) identified the conditions to differentiate cardiac fibroblasts resembling primary human quiescent cardiac fibroblasts at transcriptional, cellular, and functional levels. Of note, the maintenance of quiescent fibroblasts is fully controlled by constant inhibition of the TGF-β pathway, while fibroblast-to-myofibroblast trans-differentiation is induced by exposure to TGF-β. In this setting pirfenidone exhibits a dose-dependent inhibitory effect on TGF-β-induced fibrotic phenotype, demonstrating that, whatever the mechanism, it acts as a functional antagonist of the pro-fibrotic effects induced by TGF-β on quiescent cardiac human fibroblasts.

A further step toward an integrated cardiac model system comprising human fibroblasts and cardiomyocytes is represented by the *in-vitro* model obtained by culturing hiPSC-derived cardiomyocytes and non-myocytic cells (mostly fibroblasts) (20). Upon exposure to TGF-β, the model shows enhanced cardiac fibrotic extracellular matrix gene expression, and decreased cardiac contractile/relaxation velocity, simulating two typical pathologic responses of the fibrotic cardiac tissue. In this model pirfenidone decreased fibrotic changes induced by TGF-β and counteracted the transcription of genes encoding for collagen I, collagen III, fibronectin, and matrix metalloproteinases (MMP) type-2 (20), closely reflecting the effects of the drug observed after *in-vivo* administration.

The effect occurred at high concentrations of the drug (100–300 μM), which is, however, in line with plasma levels needed for therapeutic effects in pulmonary fibrosis. After administration of the immediate release formulation, the plasma peak concentration is approximately 10 mg/L, corresponding to 50 μM (21). Insights also came from studies in “organ-on-a-chip” mimicking human cardiac fibrosis exploiting 3D bioprinting (22). In brief, hiPSC-derived cardiomyocytes were co-seeded with human cardiac fibroblasts, quiescent, or pre-treated with TGF-β. In the latter case, the artificial tissue showed increased stiffness, due to higher collagen deposition, collagen type I to III ratio, and α-SMA-positive cells. Interestingly, BNP production was also increased, suggesting an effect on cardiomyocyte gene expression. Stiffness and collagen deposition was reduced by pirfenidone in a 3-week treatment at high concentrations (2.5 mM), along with decreased expression of fibrosis markers (periostin, CCN2). Pirfenidone also reduced BNP expression with no effects on cardiomyocyte excitation and contraction (22). Despite the evidence of pirfenidone activity against TGF-β-mediated activation of the canonical pathway SMAD *in-vitro* and consequent impairment of fibroblast activation, proliferation and collagen deposition, the target of this drug remains unclear and deserves further investigation.

### A complex crosstalk controls fibroblast activation

While TGF-β1 is the most reliable signal for MyoFib trans-differentiation, other factors can induce fibrosis, i.e., collagen deposition, by fibroblasts eventually expressing thrombospondin-4 (Thbs4) but not α-SMA (8). Vasoactive peptides, such as angiotensin II (Ang-2) and endothelin-1 (ET-1), act through the mitogen-activated protein kinase (MAPK) and Rho-associated protein kinase (ROCK) cascades. By binding to G-protein binding receptors (AT_1_ and ET_A_, respectively), these peptides are potent promoters of cardiac fibroblast activation, and their effect is potentiated by TGF-β (23). Aldosterone has been also indicated as a potent pro-fibrogenic factor (24). Despite several pieces of evidence on the antifibrotic effect of pirfenidone in animals chronically treated with Ang-2, insights into the mechanistic pathways are more uncertain. Both Ang-2 and TGF-β activate transient receptor potential (TRP) channels, among which TRPC6 seems to be a compelled step for MyoFib transdifferentiation in several tissues, including the heart (25, 26). This is particularly interesting because similar evidence came from our recent observations in satellite skeletal cells exposed to Ang-2 (27). TRPC6 is a mechanosensitive, non-selective ion channel whose expression is upregulated by TGF-β through p38/MAPK pathway (28), and pirfenidone inhibits p38 phosphorylation (29).

Atria are particularly exposed to mechanical stretch in several conditions, promoting dilation and fibrosis and thus atrial fibrillation. Of note, atrial levels of TGFβ 1 are increased in heart failure before the onset of atrial fibrillation (AF) and pirfenidone has proved to counteract fibrosis, i.e., the substrate for the occurrence and chronicization of AF (30–32). A third but no less important player is represented by oxidative stress; in particular, the production of superoxide by NADP(H) oxidase type-4 (NOX4) bursts in fibroblasts stimulated by TGF-β_1_ (33). Over-activated Nox 4 is a hallmark of several conditions, including heart failure and hypertrophic cardiomyopathy (34, 35), likely due to the membrane translocation of the p47phox subunit of the enzyme (36). That pirfenidone exerts antioxidant activity is suggested by reduction of markers of oxidative stress in patients (37) and animal models; however, it is uncertain whether this is a primary effect or the consequence of its anti-inflammatory activity. Therefore, even if the precise mechanism of pirfenidone is unknown, its antifibrotic activity may reside in the ability to interfere with upward stimuli, such as TGF-β production and macrophagic activation, and/or with several downstream steps. A general observation arises from all these studies: pirfenidone hampers TGF-β activity on fibroblasts *in vitro*, independently from the presence of inflammatory cells, cardiomyocytes or circulating stimuli. While interference with these players should not be ruled out, this observation allows us to locate one or more key target(s) of pirfenidone in the pathway between TGF-β receptor stimulation and the effectors of MyoFib transdifferentiation. In a recent study exploiting engineered cardiac tissues, pirfenidone could not counteract all pro-fibrotic transcripts and proteins upregulated by TGF-β1 stimulation (38). The authors inferred that the drug might hinder the non-canonical TGF-β1 pathway more than the canonical one; however, these data also suggest a cautious extrapolation of the mechanisms underlying the antifibrotic effect of pirfenidone from fibroblasts *in-vitro* to a more complex, multicellular setting.

### Insights from preclinical studies *in-vivo*

Pirfenidone has been proposed as an antifibrotic agent in at least three target organs: lung, kidney, and heart (23, 39). Heart failure with a reduced or preserved ejection fraction, hypertrophic cardiomyopathy, diabetic cardiomyopathy, and doxorubicin-induced cardiac injury are some of the most common diseases where extensive fibrosis represents a major culprit for contractile and electrical abnormalities. At the same time, reverse remodeling is particularly challenging and many drugs effective in animal models failed in clinical trials, such as ET-1 antagonists, anti-TNF-α, and MMP inhibitors. Drugs with hemodynamic effects, such as ACE inhibitors and antagonists of Ang-2 receptors (ARB), mineralocorticoid receptor antagonists (spironolactone, eplerenone, canrenone), do have antifibrotic effects (23), but in the context of a general cardiac reverse remodeling. Also, mechanical unload by a left ventricular assist device (LVAD) has no major impact on fibrosis and MyoFib density in patients with severe heart failure, likely due to persistent inflammation (40). Ranolazine, an antianginal drug targeting the late sodium current and calcium overload, not only blocks arrhythmogenic mechanisms but also reduces fibrosis in an adult mouse model of HCM when administrated chronically after birth (41).

Major advantages of pirfenidone reside in its specific anti-fibrotic activity, which does not affect blood pressure or electrolyte balance, and that it passed the clinical valuation for lung diseases. This drug has been tested in several animal models proxy for human cardiac disease. In myocardial hypertrophy, due to pressure overload (29), and in myocardial infarction (29, 42), pirfenidone reduced fibrosis. However, the latter case is interesting for two reasons. First, the effect was accompanied by a reduction of the arrhythmic burden, which is a major consequence of cardiac fibrosis. Second, one cannot assume that fibroblasts respond to the drug similarly, whatever their organ or sub-organ localization: scars create a border zone of the infarcted tissue and the interstitial matrix encompasses cells with different molecular and functional properties in response to stressors (8). Third, MyoFibs in the border zone are electrically coupled to cardiomyocytes and modify excitability and conduction, thus favoring reentry mechanisms (43). The capability to form connections with cardiomyocytes is typical of MyoFib, whose expression of connexin-43 is higher than in fibroblasts (44). When coupled to CMs, MyoFbs reduced the CM action potential duration and hyperpolarized the CM resting membrane potential (44, 45). Whether pirfenidone reduces connexin-43 expression and MyoFib-cardiomyocyte coupling is unknown.

## Conclusions and perspectives

Searching for new therapies against fibrosis is a priority for many ill-treated conditions such as familial hypertrophic cardiomyopathy and heart failure with preserved ejection fraction (HFpEF) (46). Whether pirfenidone might be a first-in-class drug against cardiac fibrosis is unknown: so far, the evidence raises more questions than settling sound answers and we can hardly infer efficacy and especially causal mechanisms in the clinical settings. Also, it is worth recalling that interstitial fibrosis can be boosted by different stressors and cell populations, e.g., in the infarct border zone or dilated atria (47). So far, in the interventional phase II trial PIRfenidOne in patients with heart entric and preserved lEfT entricular Ejection fraction (PIROUETTE), a 52-week treatment with pirfenidone resulted in a significant reduction of extracellular volume, a proxy of fibrosis (48). From a functional point of view, the most relevant finding consisted of a small increase of the left ventricular ejection fraction in the treatment group with respect to placebo. A subsequent, exploratory mediation analysis, aimed at gaining mechanistic insights and based on the participants' functional data and biomarkers of PIROUETTE, failed to demonstrate a causal relationship between reduced fibrosis and improved LV function; however, regression of fibrosis correlated with an increased 6-min walk test distance (49). Overall, this preliminary evidence supports the benefit of reduced myocardial fibrosis, but the underlying mechanisms deserve to be investigated by appropriately powered trials. More generally, the relevance of such a perspective is underscored by several studies focused on pirfenidone and cardiac hypertrophy/fibrosis in these years; for a more detailed summary of the evidence in the literature, the reader can refer to a recent review paper (50). Finally, pirfenidone has low potency and side effects that hamper quality of life, and, recently, the analog mefunidone (51) has been shown to exert antifibrotic activity with higher potency and more favorable pharmacokinetics.

## Author contributions

All authors listed have made a substantial, direct, and intellectual contribution to the work and approved it for publication.

## Funding

This study was supported in part by grants from the Minister of University and Research (PRIN 2017, RHYTHM-INSIGHT to EC) and from Tuscany Region (PRECAVID to EC). Human fibroblasts were kindly provided by prof. Alessandra Rossini (EURAC Research, BZ, Italy).

## Conflict of interest

The authors declare that the research was conducted in the absence of any commercial or financial relationships that could be construed as a potential conflict of interest. The handling editor AA declared a past co-authorship/collaboration doi: 10.1016/j.jacc.2020.08.031 and doi: 10.1016/j.phrs.2020.104694 with the authors EC and GB.

## Publisher's note

All claims expressed in this article are solely those of the authors and do not necessarily represent those of their affiliated organizations, or those of the publisher, the editors and the reviewers. Any product that may be evaluated in this article, or claim that may be made by its manufacturer, is not guaranteed or endorsed by the publisher.

## References

[1] Agency EM,. Esbriet (pirfenidone) hard capsules: EU summary of product characteristics. (2021). Available online at: http://www.ema.europa.eu

[2] Shi S Wu J Chen H Zeng F. Single- and multiple-dose pharmacokinetics of pirfenidone, an antifibrotic agent, in healthy Chinese volunteers. J Clin Pharmacol. (2007) 47:1268–76. 10.1177/0091270007304104 17906160

[3] Rubino CM Bhavnani SM Ambrose PG Forrest A Loutit JS. Effect of food and antacids on the pharmacokinetics of pirfenidone in older healthy adults. Pulm. Pharmacol. Ther. (2009) 22:279–85. 10.1016/j.pupt.2009.03.003 19328861

[4] FDA U,. Esbriet (pirfenidone) capsules US prescribing information. (2021). Available online at: https://www.accessdata.fda.gov/scripts/cder/daf/

[5] Macías-Barragán J Sandoval-Rodríguez A Navarro-Partida J Armendáriz-Borunda J. The multifaceted role of pirfenidone and its novel targets. Fibrogenesis Tissue Repair. (2010) 3:1–11. 10.1186/1755-1536-3-16 20809935 PMC2944211

[6] Janka-Zires M Almeda-Valdes P Uribe-Wiechers AC Juárez-Comboni SC López-Gutiérrez J Escobar-Jiménez JJ. Topical administration of pirfenidone increases healing of chronic diabetic foot ulcers: a randomized crossover study. J Diabetes Res. (2016) 2016:7340641. 10.1155/2016/7340641 27478849 PMC4958428

[7] Graziani F Lillo R Crea F. Rationale for the use of pirfenidone in heart failure with preserved ejection fraction. Front Cardiovasc Med. (2021) 8:678530. 10.3389/fcvm.2021.678530 33969025 PMC8100203

[8] McLellan MA Skelly DA Dona MSI Squiers GT Farrugia GE Gaynor TL. High-resolution transcriptomic profiling of the heart during chronic stress reveals cellular drivers of cardiac fibrosis and hypertrophy. Circulation. (2020) 1448–63. 10.1161/CIRCULATIONAHA.119.045115 PMC7547893 32795101

[9] Ivanova A Signore M Caro N Greene NDE Copp AJ Martinez-Barbera JP. *In vivo* genetic ablation by Cre-mediated expression of diphtheria toxin fragment A. Genesis. (2005) 43:129–35. 10.1002/gene.20162 16267821 PMC2233880

[10] Wynn TA Ramalingam TR. Mechanisms of fibrosis: Therapeutic translation for fibrotic disease. Nat Med. (2012) 18:1028–40. 10.1038/nm.2807 22772564 PMC3405917

[11] Tallquist MD Molkentin JD. Redefining the identity of cardiac fibroblasts. Nature Rev Cardiol. (2017) 14:484–91. 10.1038/nrcardio.2017.57 28436487 PMC6329009

[12] Meilhac SM Buckingham ME. The deployment of cell lineages that form the mammalian heart. Nat Rev Cardiol. (2018) 15:705–24. 10.1038/s41569-018-0086-9 30266935

[13] Frantz S Hu K Adamek A Wolf J Sallam A Maier SK. Transforming growth factor beta inhibition increases mortality and left ventricular dilatation after myocardial infarction. Basic Res Cardiol. (2008) 103:485–92. 10.1007/s00395-008-0739-7 18651091

[14] Leask A. (2015). Getting to the heart of the matter: new insights into cardiac fibrosis. Circ Res. 116:1269–76. 10.1161/CIRCRESAHA.116.305381 25814687

[15] Shi Q Li X Bai Y Cui C Li J. *In vitro* effects of pirfenidone on cardiac fibroblasts: proliferation, myofibroblast differentiation, migration and cytokine secretion. PLoS ONE. (2011) 6:e28134. 10.1371/journal.pone.0028134 22132230 PMC3223242

[16] Santiago JJ Dangerfield AL Rattan SG Bathe KL Cunnington RH Raizman JE. Cardiac fibroblast to myofibroblast differentiation *in vivo* and *in vitro*: expression of focal adhesion components in neonatal and adult rat ventricular myofibroblasts. Dev Dynam. (2010) 239:1573–84. 10.1002/dvdy.22280 20503355

[17] Landry NM Rattan SG Dixon IMC. An improved method of maintaining primary murine cardiac fibroblasts in two-dimensional cell culture. Scientific Rep. (2019) 9. 10.1038/s41598-019-49285-9 PMC6733858 31501457

[18] Zhang H Tian L Shen M Tu C Wu H Gu M. Generation of quiescent cardiac fibroblasts from human induced pluripotent stem cells for *in vitro* modeling of cardiac fibrosis. Circ Res. (2019) 125:552–66. 10.1161/CIRCRESAHA.119.315491 31288631 PMC6768436

[19] Zhang H Shen M Wu JC. Generation of quiescent cardiac fibroblasts derived from human induced pluripotent stem cells. Methods Mol Biol. (2020) 2454:109–15. 10.1007/7651_2020_300 32671814 PMC9667911

[20] Iseoka H Miyagawa S Sakai Y Sawa Y. Cardiac fibrosis models using human induced pluripotent stem cell-derived cardiac tissues allow anti-fibrotic drug screening *in vitro*. Stem Cell Res. (2021) 54:102420. 10.1016/j.scr.2021.102420 34126557

[21] Barranco-Garduño LM Buendía-Roldan I Rodriguez JJ González-Ramírez R Cervantes-Nevárez AN Neri-Salvador JC. Pharmacokinetic evaluation of two pirfenidone formulations in patients with idiopathic pulmonary fibrosis and chronic hypersensitivity pneumonitis. Heliyon. (2020) 6:e05279. 10.1016/j.heliyon.2020.e05279 33163646 PMC7610245

[22] Mastikhina O Moon BU Williams K Hatkar R Gustafson D Mourad O. Human cardiac fibrosis-on-a-chip model recapitulates disease hallmarks and can serve as a platform for drug testing. Biomaterials. (2020) 233:119741. 10.1016/j.biomaterials.2019.119741 31927251

[23] Rockey DC Darwin Bell P Hill JA. Fibrosis-a common pathway to organ injury and failure. N Engl J Med. (2015) 372:1138–49. 10.1056/NEJMra1300575 25785971

[24] Wang B Ding W Zhang M Li H Gu Y. Rapamycin attenuates aldosterone-induced tubulointerstitial inflammation and fibrosis. Cell Physiol Biochem. (2015) 35:116–25. 10.1159/000369680 25547416

[25] Davis J Bur AR Davis GF Birnbaumer L Molkentin JD. A TRPC6-dependent pathway for myofibroblast transdifferentiation and wound healing *in vivo*. Dev cell. (2012) 23:705–15. 10.1016/j.devcel.2012.08.017 23022034 PMC3505601

[26] Thodeti CK Paruchuri S Meszaros JG. A TRP to cardiac fibroblast differentiation. Channels. (2013) 7:211–4. 10.4161/chan.24328 23511028 PMC3710348

[27] Laurino A Spinelli V Gencarelli M Balducci V Dini L Diolaiuti L. Angiotensin-ii drives human satellite cells toward hypertrophy and myofibroblast trans-differentiation by two independent pathways. Int J Mol Sci. (2019) 20:4912. 10.3390/ijms20194912 31623362 PMC6801484

[28] Stewart L Turner NA. Channelling the force to reprogram the matrix: mechanosensitive ion channels in cardiac fibroblasts. Cells. (2021) 10:990. 10.3390/cells10050990 33922466 PMC8145896

[29] Yamagami K Oka T Wang Q Ishizu T Lee JK Miwa K. Pirfenidone exhibits cardioprotective effects by regulating myocardial fibrosis and vascular permeability in pressure-overloaded hearts. Am J Physiol Heart Circ Physiol. (2015) 309:H512–22. 10.1152/ajpheart.00137.2015 26055790

[30] Burstein B Nattel S. Atrial fibrosis: mechanisms and clinical relevance in atrial fibrillation. J Am Coll Cardiol. (2008) 51:802–9. 10.1016/j.jacc.2007.09.064 18294563

[31] Lee KW Everett Iv TH Rahmutula D Guerra JM Wilson E Ding C. Pirfenidone prevents the development of a vulnerable substrate for atrial fibrillation in a canine model of heart failure. Circulation. (2006) 114:1703–12. 10.1161/CIRCULATIONAHA.106.624320 17030685 PMC2129103

[32] Rahmutula D Marcus GM Wilson EE Ding CH Xiao Y Paquet AC. Molecular basis of selective atrial fibrosis due to overexpression of transforming growth factor-β1. Cardiovasc Res. (2013) 99:769–79. 10.1093/cvr/cvt074 23612580 PMC3746950

[33] Cucoranu I Clempus R Dikalova A Phelan PJ Ariyan S Dikalov S. NAD(P)H oxidase 4 mediates transforming growth factor-β1-induced differentiation of cardiac fibroblasts into myofibroblasts. Circ Res. (2005) 97:900–7. 10.1161/01.RES.0000187457.24338.3D 16179589

[34] Santini L Palandri C Nediani C Cerbai E Coppini R. Modelling genetic diseases for drug development: Hypertrophic cardiomyopathy. Pharmacol Res. (2020) 160:105176. 10.1016/j.phrs.2020.105176 32871247

[35] Nediani C Borchi E Giordano C Baruzzo S Ponziani V Sebastiani M. NADPH oxidase-dependent redox signaling in human heart failure: relationship between the left and right ventricle. J Mol Cell Cardiol. (2007) 42:826–34. 10.1016/j.yjmcc.2007.01.009 17346742

[36] Nediani C Raimondi L Borchi E Cerbai E. Nitric oxide/reactive oxygen species generation and nitroso/redox imbalance in heart failure: from molecular mechanisms to therapeutic implications. Antioxid Redox Signal. (2011) 14:289–331. 10.1089/ars.2010.3198 20624031

[37] Fois AG Sotgiu E Scano V Negri S Mellino S Zinellu E. Effects of pirfenidone and nintedanib on markers of systemic oxidative stress and inflammation in patients with idiopathic pulmonary fibrosis: a preliminary report. Antioxidants. (2020) 9:1–15. 10.3390/antiox9111064 33143144 PMC7692317

[38] Bracco Gartner TCL Crnko S Leiteris L van Adrichem I van Laake LW Bouten CVC. Pirfenidone has anti-fibrotic effects in a tissue-engineered model of human cardiac fibrosis. Front Cardiovasc Med. (2022) 9:854314. 10.3389/fcvm.2022.854314 35360018 PMC8963358

[39] Aimo A Cerbai E Bartolucci G Adamo L Barison A Lo Surdo G. Pirfenidone is a cardioprotective drug: MECHANISMS of action and preclinical evidence. Pharmacol Res. (2020) 155:104694. 10.1016/j.phrs.2020.104694 32061664

[40] Farris SD Don C Helterline D Costa C Plummer T Steffes S. Cell-specific pathways supporting persistent fibrosis in heart failure. J Am Coll Cardiol. (2017) 70:344–54. 10.1016/j.jacc.2017.05.040 28705316

[41] Coppini R Mazzoni L Ferrantini C Gentile F Pioner JM Laurino A. Ranolazine prevents phenotype development in a mouse model of hypertrophic cardiomyopathy. Circulation. (2017) 10e003565. 10.1161/CIRCHEARTFAILURE.116.003565 PMC6284403 28255011

[42] Nguyen DT Ding C Wilson E Marcus GM Olgin JE. Pirfenidone mitigates left ventricular fibrosis and dysfunction after myocardial infarction and reduces arrhythmias. Heart Rhythm. (2010) 7:1438–45. 10.1016/j.hrthm.2010.04.030 20433946

[43] Rubart M Tao W Lu XL Conway SJ Reuter SP Lin SF. Electrical coupling between ventricular myocytes and myofibroblasts in the infarcted mouse heart. Cardiovasc Res. (2018) 114:389–400. 10.1093/cvr/cvx163 29016731 PMC6018934

[44] Nagaraju CK Dries E Gilbert G Abdesselem M Wang N Amoni M. Myofibroblast modulation of cardiac myocyte structure and function. Sci Rep. (2019) 9:8879. 10.1038/s41598-019-45078-2 31222006 PMC6586929

[45] Nagaraju CK Robinson EL Abdesselem M Trenson S Dries E Gilbert G. Myofibroblast Phenotype and Reversibility of Fibrosis in Patients With End-Stage Heart Failure. J Am Coll Cardiol. (2019) 73:2267–82. 10.1016/j.jacc.2019.02.049 31072570

[46] Brown RD Ambler SK Mitchell MD Long CS. The cardiac fibroblast: therapeutic target in myocardial remodeling and failure. Annu. Rev. Pharmacol. Toxicol. (2005) 45:657–87. 10.1146/annurev.pharmtox.45.120403.095802 15822192

[47] Litvinuková M Talavera-López C Maatz H Reichart D Worth CL Lindberg EL. Cells of the adult human heart. Nature. (2020) 588:466–72. 10.1038/s41586-020-2797-4 32971526 PMC7681775

[48] Lewis GA Schelbert EB Naish JH Bedson E Dodd S Eccleson H. Pirfenidone in heart failure with preserved ejection fraction-rationale and design of the PIROUETTE trial. Cardiovasc Drugs Ther. (2019) 33:461–70. 10.1007/s10557-019-06876-y 31069575 PMC6689029

[49] Lewis GA Rosala-Hallas A Dodd S Schelbert EB Williams SG Cunnington C. Impact of myocardial fibrosis on cardiovascular structure, function and functional status in heart failure with preserved ejection fraction. J Cardiovasc Transl Res. (2022). 10.1007/s12265-022-10264-7. [Epub ahead of print]. PMC9722869 35790651

[50] Aimo A Spitaleri G Panichella G Lupón J Emdin M Bayes-Genis A. Pirfenidone as a novel cardiac protective treatment. Heart Fail Rev. (2022) 27:525–32. 10.1007/s10741-021-10175-w 34671871 PMC8898227

[51] Luo N Sun M Han X Li L Wang L Cheng Z. Preclinical metabolic characterization of mefunidone, a novel anti-renal fibrosis drug. Life Sciences. (2021) 280. 10.1016/j.lfs.2021.119666 34087279

